# *Aggregatibacter actinomycetemcomitans* pneumonia mimicking lung cancer in a previously healthy 12-year-old child from Saudi Arabia: a case report

**DOI:** 10.11604/pamj.2020.36.89.21996

**Published:** 2020-06-15

**Authors:** Abdullah Al-Nafeesah

**Affiliations:** 1 Department of Pediatrics, Unaizah College of Medicine and Medical Sciences, Qassim University, Kingdom of Saudi Arabia

**Keywords:** *Aggregatibacter actinomycetemcomitans*, pneumonia, children, Saudi Arabia

## Abstract

*Aggregatibacter actinomycetemcomitans* formely known as *Actinobacillus actinomycetemcomitans* is a known part of the normal oral flora. It can sometimes cause oral or rarely extra-oral infections secondary to hematogenous extension or aspiration. It is a rare cause of invasive pneumonia. It can resemble tuberculosis or lung cancer in its presentation. Making the diagnosis in such case is crucial for better management that usually require tissue biopsy. We report a case of *Aggregatibacter actinomycetemcomitans* invasive pneumonia in a 12-year-old previously healthy boy from Saudi Arabia. After a prolonged course of antibiotics, clinical and radiological follow up showed complete resolution of the infection.

## Introduction

Chronic lung infections sometimes poses a diagnostic dilemma and can mimic tuberculosis or lung cancer [1]. All can present with unspecific signs and symptoms like cough, fever and weight loss. Radiological imaging usually is not conclusive in reaching a diagnosis for which tissue biopsy is indicated. *Aggregatibacter actinomycetemcomitans* is a known part of the normal oral flora. It can sometimes causes sever oral and rarely extra-oral infections like infective endocarditis, lung infection, brain abscess, head and neck, osteo-articular, soft tissue and urethral infections [2, 3]. Lung infection caused by this organism is very rare in children. We present up to our knowledge the first case in English medical literature of *Aggregatibacter actinomycetemcomitans* lung infection in Saudi Arabian children and a review of the other published similar cases in English medical literature.

## Patient and observation

**Figure 1:**
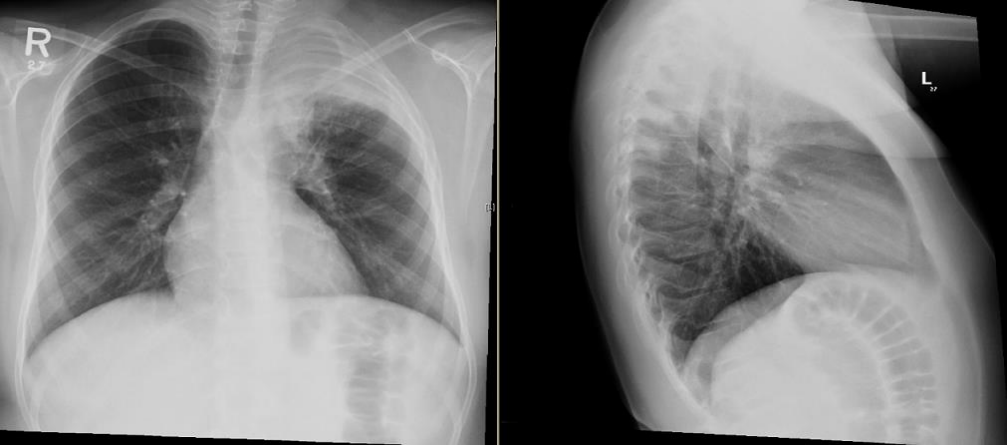
chest X-ray showed left perihilar and upper lobe opacity

**Figure 2:**
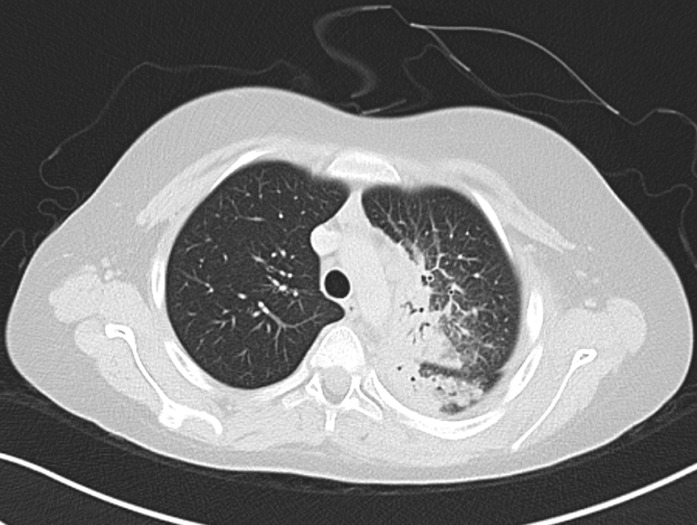
CT chest showed multiple left hilar lymph nodes with collapse of the left upper lobe and multiple pulmonary nodules with cavitation

**Figure 3:**
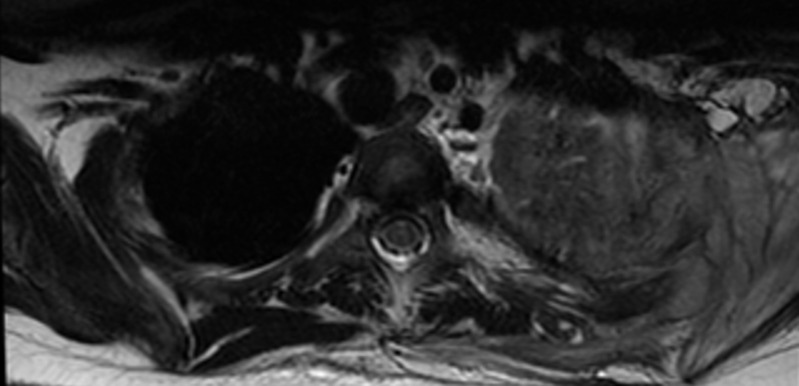
magnetic resonance imaging showed destructive left apical /supraclavicular enhancing lesion, which invades the peripheral brachial plexopathy

A 12-year old boy healthy before referred to our hospital due to left apical lung aggressive destructive lesion for further evaluation. The problem started 2 months prior to presentation with left shoulder pain that progress with time. There was a history of mild cough with no history of fever. There was a history of weight loss of 5 kilograms. No other symptoms were noted. Upon examination at the hospital he was afebrile and maintain hemodynamics. He was active, not in respiratory distress with limited active and passive left shoulder movement to around 45 degree. There was minimal tenderness over the left clavicle medially with no overlying skin inflammatory changes or chest wall swelling. Chest examination showed diminished air entry in the left upper zone. Mouth examination were normal. There was no organomegally or systemic lymphadenopathy. With this presentation of chronic left lung aggressive destructive lesion, weight loss and absence of fever we kept malignancy on the top differential diagnosis that require further evaluation. His complete blood count showed white blood count 10.9x10g/L, hemoglobin 103gm/L and platelet 646x10g/L. Erythrocyte sedimentation rate was 50 mm/hr and C reactive protein 179mg/L. Chest X-ray showed left perihilar and upper lobe opacity (Figure 1). CT chest showed multiple left hilar lymph nodes with collapse of the left upper lobe and multiple pulmonary nodules with cavitation. There was inflammatory changes around the left rip with evidence of bone destruction and soft tissue inflammation (Figure 2). Magnetic resonance imaging showed destructive left apical /supraclavicular enhancing lesion, which invades the peripheral brachial plexopathy (Figure 3). CT guided transthoracic tissue biopsy showed chronic inflammatory cells and the culture showed light growth of *Aggregatibacter actinomycetemcomitans*. The patient started on intravenous cefotaxime and clindamycin for 1 month then he completed 4 months with oral Cefprozil and Metronodazole. The total duration of therapy was 5 months with complete clinical and radiological resolution.

## Discussion

*Actinobacillus actinomycetemcomitans* named initially by Klinger in 1912 [4]. At that time, it was not known to cause diseases to human being. In 1951, Thjoha and Sydne reported the first infection caused by this organism to human [5] as ‘1umpy jaw’, after that other organs involvement were reported. Later on, *Actinobacillus actinomycetemcomitans* has been reported in cases of vertebral osteomyelitis, brain abscess, meningitis, thyroid abscess, urinary tract infection, synovitis and pneumonia [4]. After reviewing the English medical literature, we found reports of eight cases of *Aggregatibacter actinomycetemcomitans* pneumonia in children [6-12] our patient is the first case in the Kingdom of Saudi Arabia. Predisposing dental disease were found in some cases [6, 8, 9, 13]. Fever were high only in three of the cases possessing a diagnostic dilemma. As the presentation is nonspecific for an infectious process, malignancy has been suspected in these cases especially with the radiological findings. Other differential diagnosis that was considered in this case is Mycobacterium tuberculosis infection. Thoracic actinomycosis can resemble pulmonary tuberculosis, because both infections might present with infiltrate surrounding cavitary lesion [14]. After appropriate tissue sampling for routine culture or using the new molecular modalities like PCR diagnosis can be reached [12]. Management plan depends on stabilizing the patient and starting the appropriate antimicrobial regimen based on the susceptibility tests. Usually *Aggregatibacter actinomycetemcomitans* susceptible to fluoroquinolones, cephalosporines, mezlocillin, Trimethoprim-sulfamethoxazole, aminoglicosides, chloramphenicol, azithromycin and tetracyclin. Patterns of resistant are variable to penicillin, amoxicillin, clindamycin, and metronidazole [15]. In vitro susceptibility to clindamycin, vancomycin and erythromycin were found. However, Duration of treatment is uncertain but overall extended therapy is recommended [12]. It depends on how extensive is the infection and how far tissue were involved. It has been mentioned in the literature that 3-12 months duration is satisfactory [8].

## Conclusion

*Actinobacillus actinomycetemcomitans* pneumonia is very rare in children. It can be mistaken as Tuberculosis or malignancy. Diagnosis need high index of suspicion and tissue biopsy. Duration of treatment depends on the clinical and radiological response.
